# Methodological considerations on segmenting rhabdomyosarcoma with diffusion-weighted imaging—What can we do better?

**DOI:** 10.1186/s13244-022-01351-z

**Published:** 2023-01-31

**Authors:** Cyrano Chatziantoniou, Reineke A. Schoot, Roelof van Ewijk, Rick R. van Rijn, Simone A. J. ter Horst, Johannes H. M. Merks, Alexander Leemans, Alberto De Luca

**Affiliations:** 1 Image Sciences Institute, UMC Utrecht, Utrecht, The Netherlands; 2 Princess Máxima Center for Pediatric Oncology, Utrecht, The Netherlands; 3 Department of Neurology, UMC Utrecht Brain Center, UMCUtrecht, Utrecht, The Netherlands; 4 Department of Radiology and Nuclear Medicine, Amsterdam UMC Location University of Amsterdam, Amsterdam, The Netherlands; 5 Department of Radiology and Nuclear Medicine, Wilhelmina Children’s Hospital UMC Utrecht, Utrecht, The Netherlands

**Keywords:** Sarcoma, Magnetic resonance imaging, Rhabdomyosarcoma

## Abstract

**Purpose:**

Diffusion-weighted MRI is a promising technique to monitor response to treatment in pediatric rhabdomyosarcoma. However, its validation in clinical practice remains challenging. This study aims to investigate how the tumor segmentation strategy can affect the apparent diffusion coefficient (ADC) measured in pediatric rhabdomyosarcoma.

**Materials and methods:**

A literature review was performed in PubMed using search terms relating to MRI and sarcomas to identify commonly applied segmentation strategies. Seventy-six articles were included, and their presented segmentation methods were evaluated. Commonly reported segmentation strategies were then evaluated on diffusion-weighted imaging of five pediatric rhabdomyosarcoma patients to assess their impact on ADC.

**Results:**

We found that studies applied different segmentation strategies to define the shape of the region of interest (ROI)(outline 60%, circular ROI 27%), to define the segmentation volume (2D 44%, multislice 9%, 3D 21%), and to define the segmentation area (excludes edge 7%, excludes other region 19%, specific area 27%, whole tumor 48%). In addition, details of the segmentation strategy are often unreported. When implementing and comparing these strategies on in-house data, we found that excluding necrotic, cystic, and hemorrhagic areas from segmentations resulted in on average 5.6% lower mean ADC. Additionally, the slice location used in 2D segmentation methods could affect ADC by as much as 66%.

**Conclusion:**

Diffusion-weighted MRI studies in pediatric sarcoma currently employ a variety of segmentation methods. Our study shows that different segmentation strategies can result in vastly different ADC measurements, highlighting the importance to further investigate and standardize segmentation.

**Supplementary Information:**

The online version contains supplementary material available at 10.1186/s13244-022-01351-z.

## Key points

- Strategies for segmenting sarcoma tumors vary widely throughout literature.
- Details of the segmentation strategy are often not reported.
- Including necrotic or cystic areas in the segmentation affects diffusion measurements.
- Varying the slice of a single-slice segmentation can drastically impact diffusion measurements.

**Supplementary Information:**

The online version contains supplementary material available at 10.1186/s13244-022-01351-z.

## Introduction

Rhabdomyosarcoma is an aggressive tumor that can arise in any part of the body and is thought to stem from primitive mesenchymal cells. Rhabdomyosarcoma is the most common soft tissue sarcoma in children and adolescents and requires multimodal treatment including chemotherapy and local therapy (surgery and/or radiotherapy). Although therapy advances have been made over het last decades, important improvements in therapy are needed as patients with high risk disease have suboptimal survival. Markers of response for pediatric rhabdomyosarcoma are urgently needed for the evaluation of new agents in clinical trials, and to support (de)intensification of treatment for individual patients. Since rhabdomyosarcoma is a rare disease (with an incidence of about 4.5 cases per million children [1]), collaborative, international multicenter studies are necessary to achieve a sample size that is sufficient to identify and validate biomarkers. To date, the change in tumor size is used to evaluate response and to support decisions to continue or change (systemic) treatment. However, in the past decade, several studies have challenged the prognostic value of change in size in relation to outcome [2–7]. Additionally, size response has been criticized for its moderate reproducibility between observers [8]. Therefore, there is a clear need for a new early response biomarker that can impact prognosis. It has been suggested that diffusion-weighted MRI (DWI)-based radiomics may provide such a prognostic marker.

Previous exploratory studies have suggested the existence of a relation between water diffusion in the tumor and tumor response [9, 10]. The general theory behind this relation is that a decrease in tumor cellularity due to therapy increases the rate of water diffusion, leading to a higher quantified diffusion-weighted MRI parameter, the apparent diffusion coefficient (ADC) [11, 12]. Similar studies have examined this relation for other tumors, with mixed results [11, 13, 14]. To date, no study has reliably demonstrated whether ADC could predict response to treatment in rhabdomyosarcoma with sufficient accuracy [9].

An important aspect of DWI-based radiomics is the definition of the region of interest (ROI) used to measure the ADC. To date there are no international guidelines describing how these regions should be defined, and as such different strategies are being used [5]. Although apparently straightforward, a segmentation strategy consists of many methodological choices and parameters. These include, for example, the number of slices included in the analysis, whether to draw a polygonal outline following the tumor border or a circular ROI, and which part(s) of the tumor to include. Currently, despite the ubiquitous use of segmentations to measure the ADC in tumors, little is known on how these choices affect the ADC measurements.

To address this knowledge gap, we explored how segmentation strategies differ in recent studies relating to sarcomas. To this end, we first performed a literature review to gather which methods are most commonly applied for tumor segmentation in sarcoma imaging. Second, we conducted a pilot study to investigate the impact of the choice of a selected number of segmentation strategies on the ADC measured in pediatric rhabdomyosarcoma.

## Methods

Our study consists of two main parts. In the first part, we performed a literature review on tumor segmentation methods applied in sarcoma research. In the second part, we explored the impact of the choice of a number of segmentation strategies on the ADC measured in a representative sample of pediatric rhabdomyosarcoma cases.

## Literature review

### Literature search

**Fig. 1:**
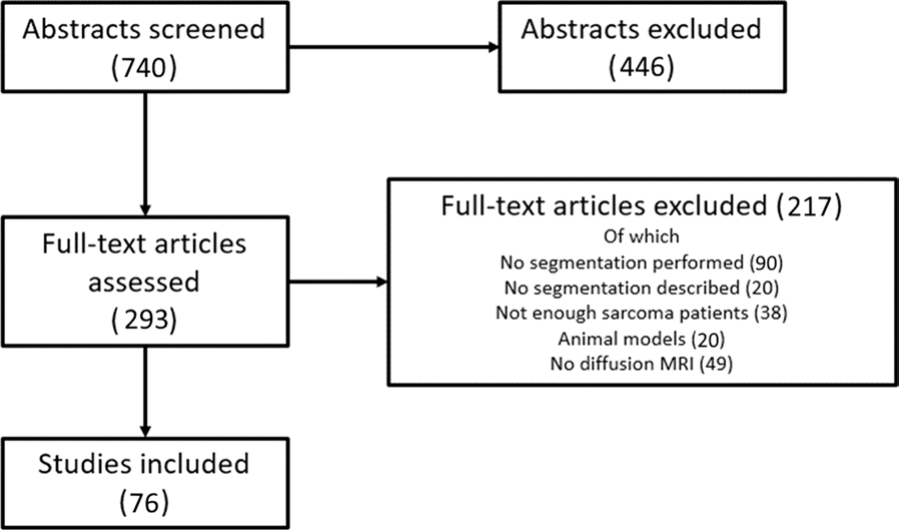
From the 740 studies in the search, 446 were excluded based on the title or abstract. Articles were excluded if they did not mention or imply MRI and segmentation of a sarcoma. Next, out of the 293 remaining articles, 217 were excluded based on the full text. Articles were excluded if no DWI was performed, if no details on segmentation were present, or if MRI was only performed on animals. Additionally, articles were excluded if they included less than 10 sarcoma patients and those patients constituted less than 50% of the total patient population

A literature search was performed using PubMed on 24-03-2022. The search included terms on MRI and sarcomas (see Additional file 1). Publication language was restricted to English. Publication status was not restricted. Articles were excluded if they did not mention or imply MRI and segmentation of sarcomas in the title or abstract. Based on the full text, articles were furthermore excluded if DWI was not performed, if no details on segmentation methods were present, or if MRI was only done on animal models. Additionally, given the focus of this work on sarcomas, articles were excluded if they reported on less than 10 sarcoma patients and if those patients constituted less than 50% of the total patient population. An overview of the selection is shown in Fig. 1.

### Segmentation strategy details

For the remaining 76 articles, details on the segmentation of the tumors were collected. Two papers used multiple methods. These have all been included as separate strategies.

The details of interest were the image modality (diffusion, structural), the segmentation method (outline, circular ROI), the segmentation area (whole tumor, excludes edge, excludes other, specific area), and the segmentation volume (2D, multislice, 3D). Here, we define a multislice segmentation as a segmentation on multiple, but not all, slices where a tumor is present. Whenever a paper did not explicitly state the individual aspects and did not provide any other information from which they could be determined, that aspect was noted as ‘unclear.’ Some articles excluded both the edge and other areas. These were then both counted. We lastly recorded whether any aspects of the segmentation strategies were justified with a reference to another study.

### Segmentation analysis

In order to quantitatively compare the effects of different segmentation strategies on ADC measurements, we selected six segmentation methods that best represented the majority of the strategies found in the literature review (for details, see literature review results). They were then used to segment DWI images of pediatric rhabdomyosarcoma patients. In order to gain some insight into how the ADC distribution might vary throughout the tumor, as well as to gauge the robustness of single-slice segmentations, these segmentations were applied on a slice nearby the initial one.

### Patients

Five pediatric rhabdomyosarcoma patients were selected from a cohort treated at Princess Máxima Center (Utrecht, the Netherlands). Explicit consent was obtained for all patients. Patients were selected to represent different tumor sizes, as well as different amounts of necrotic and cystic areas present. Patients were all male, 1–17 years of age, with either an embryonal (n = 3, fusion negative), alveolar (n = 1, PAX7-FOXO1 positive), or sclerosing rhabdomyosarcoma (n = 1). Three primary tumors were located in the bladder, one in the lower extremity and one in the upper extremity. Patients were all treated and imaged according to the E*p*SSG RMS 2005 protocol [15]. Only imaging performed at diagnosis (i.e., before treatment) was used for this analysis.

### Image analysis

Image segmentation was performed by two pediatric radiologists (S.H. and R.R.) with 10 and 18 years of experience in pediatric oncology. All segmentations were subsequently reviewed in a consensus reading with both radiologists. Prior to segmenting, the slice with the largest cross-sectional tumor area was manually identified.

The regions of interest were annotated via an in-house developed program that allowed the radiologists to place a polygon on the outline of the tumors and circles within the tumor. The polygon’s edges could be moved after initial placement to refine the segmentation when needed. The segmentation was performed on ADC maps, and raters could refer to corresponding T1, T1 + Gadolinium, or T2 images during the process for additional information.

For each patient, six ROIs were drawn. These were based on the most frequently occurring strategies identified in our review. The ROIs were drawn on the axial slice with the largest tumor area. The raters first drew the whole outline and then repeated the outline slightly (2 mm) inside the outer edge. Next, they drew an additional outline along the edges of any necrotic, cystic, or hemorrhagic components. Any solid areas within these components were included in this outline. Lastly, they drew the large circular ROI as well as the smaller circular ROIs. The small circular ROIs were placed in three areas with a high degree of diffusion restriction, which was identified visually by the radiologists based on the ADC images. Segmentations could be hidden in the program to allow the raters to draw multiple ROIs on a single slice without it becoming too cluttered. Another set of six ROIs was then drawn in the exact same manner on a slice two steps down in the caudal direction.

### Statistical analysis

To evaluate whether different segmentation methods lead to different ADC distributions within the ROIs, a two-sample Kolmogorov–Smirnov test was performed between the results of any pair of methods. This test was chosen based on its ability to pairwise compare distributions without making prior assumptions on their shape.

Another two-sample Kolmogorov–Smirnov test was applied to ADC values measured on different slices for all strategies. This was done to determine whether the choice of slice used in a single-slice annotation might lead to a difference in ADC distribution.

## Results

### Literature review

The literature search yielded 740 results. From these, 446 were excluded based on the title or abstract. Out of the 293 remaining articles, 217 were excluded based on the full text. Twenty articles were excluded based on full text due to unclear reporting; they did not report any details at all on the segmentation strategy, while segmentation was implied or stated to be performed. In total 76 articles were included. An overview of the included papers is shown in Fig. 1. Details on the included articles are listed in Table 1. The patients included in the 76 articles were: rhabdomyosarcoma 6.6% (5 articles), out of which 5.3% (4 articles) pediatric rhabdomyosarcoma; osteosarcoma 21.1% (16 articles); uterine sarcoma 11.8% (9 articles); hepatic angiosarcoma 1.3% (1 article); leiomyosarcoma 6.6% (5 articles); endometrial stromal sarcoma 1.3% (1 article); pulmonary artery sarcoma 1.3% (1 article); chondrosarcoma 3.3% (2 articles); and multiple soft tissue sarcomas 47.4% (36 articles).

**Table 1:** The selected studies included patients with either rhabdomyosarcoma (RMS), osteosarcoma (OS), uterine sarcoma (US), chondrosarcoma (CS), leiomyosarcoma (LMS), pulmonary artery sarcoma (PAS), hepatic angiosarcoma (HA), endometrial stromal sarcoma (ESS), or multiple (soft tissue) sarcomas. Non-sarcoma patients not shown. The studies performed segmentation for either response assessment (RA), differentiation (diff), or other purposes. Studies marked with an asterisk (*) included multiple segmentation strategies

First Author	Year	Sarcoma type	Patients	Reason	Volume	Process	Method	Image modality	References
Abdel Razek	2013	Multiple	17	Diff	2D	Manual	Outline	Diffusion	[22]
Aktas	2021	Multiple	42	RA	Unclear	Manual	Unclear	Structural	[23]
Albalawi	2019	RMS	26	Other	2D	Manual	Unclear	Diffusion	[24]
Alsharief	2019	Multiple	21	Diff	2D	Manual	Circular ROI	Diffusion	[25]
Ashikyan	2021	Multiple	15	other	Unclear	Manual	Outline	Diffusion	[26]
Asmar	2020	Multiple	15	RA	2D	Manual	Outline	Structural	[27]
Baidya Kayal	2019	OS	40	RA	3D	Manual	Outline	Diffusion	[28]
Bajpai	2009	OS	31	Other	Unclear	Manual	Outline	Diffusion	[29]
Bajpai	2011	OS	31	RA	MS	Manual	Unclear	Diffusion	[30]
Banerjee	2018	RMS	21	Diff	3D	Semi-Automated	Outline	Diffusion	[31]
Baunin	2012	OS	15	Diff	2D	Manual	Unclear	Diffusion	[32]
Bi	2018	US	60	Diff	MS	Manual	Circular ROI	Diffusion	[33]
Bi	2020	US	71	Diff	2D	Manual	Circular ROI	Diffusion	[34]
Bologna	2017	Multiple	18	Other	3D	Manual	Outline	Diffusion	[35]
Bruegel	2013	HE	7	Other	2D	Manual	Circular ROI	Diffusion	[36]
Byun	2013	OS	28	RA	2D	Manual	Outline	Diffusion	[37]
Chhabra	2019	Multiple	43	Other	2D	Manual	Circular ROI	Diffusion	[38]
Chodyla	2021	Multiple	37	diff	3D	Manual	Outline	Diffusion	[39]
Chodyla	2021	Multiple	52	RA	3D	Manual	Outline	Diffusion	[40]
Corino	2018	Multiple	19	Diff	3D	Manual	Outline	Diffusion	[41]
Degnan	2018	Multiple	18	RA	2D	Manual	Outline	Diffusion	[42]
Del Grande	2014	Multiple	37	Other	Unclear	Manual	Unclear	Diffusion	[43]
Dudeck	2008	Multiple	23	RA	MS	Manual	Circular ROI	Diffusion	[44]
Einarsdóttir	2004	Multiple	13	Other	2D	Manual	Outline	Diffusion	[45]
Gao	2017	RMS	6	RA	Unclear	Manual	Unclear	Diffusion	[46]
Gao	2021	Multiple	30	RA	Unclear	Manual	Outline	Diffusion	[47]
Gerges	2018	LMS	17	Diff	3D	Manual	Outline	Diffusion	[48]
Habre	2021	OS	26	diff	2D	Manual	Outline	Structural	[49]
Hao	2021	OS	34	RA	Unclear	Manual	Outline	Diffusion	[50]
Hélage	2021	US	50	diff	Unclear	Manual	Circular ROI	Diffusion	[51]
Hong	2020	Multiple	12	diff	3D	Manual	Outline	Diffusion	[52]
Huang	2019	US	20	Diff	2D	Manual	Outline	Unclear	[53]
Ioannidis	2019	Multiple	22	Other	3D	Manual	Circular ROI	Diffusion	[54]
Kralik	2018	RMS	12	Diff	2D	Manual	Outline	Diffusion	[55]
Lee	2020	Multiple	36	Other	2D	Manual	Outline	Structural	[56]
Lee*	2020	OS	35	RA	Multiple	Manual	Multiple	Diffusion	[57]
Li	2017	LMS	16	Diff	MS	Manual	Circular ROI	Diffusion	[58]
Li	2017	ESS	15	Other	MS	Manual	Circular ROI	Diffusion	[59]
Li	2021	Multiple	40	other	2D	Manual	Outline	Diffusion	[60]
Li	2021	Multiple	34	other	2D	Manual	Outline	Diffusion	[61]
Liu	2017	PAS	6	Diff	2D	Manual	Circular ROI	Diffusion	[62]
Liu	2019	OS	29	RA	2D	Manual	Outline	Diffusion	[63]
Manikis	2021	Multiple	28	diff	3D	Manual	Outline	Diffusion	[64]
Müller	2016	CS	32	Diff	2D	Manual	Outline	Diffusion	[65]
Nakagawa	2019	US	11	Diff	3D	Manual	Outline	Structural	[66]
Nakagawa	2019	US	30	Diff	2D	Manual	Outline	Structural	[67]
Oka	2010	OS	22	RA	2D	Manual	Circular ROI	Diffusion	[68]
Orsatti	2021	Multiple	13	RA	3D	Manual	Outline	Diffusion	[69]
Oztürk	2021	Multiple	18	other	Unclear	Manual	Circular ROI	Diffusion	[70]
Parlak	2021	Multiple	35	diff	Unclear	Manual	Outline	Diffusion	[71]
Pourmehdi Lahiji	2019	RMS	21	RA	2D	Manual	Outline	Diffusion	[9]
Rio	2019	LMS	20	Diff	2D	Manual	Circular ROI	Diffusion	[72]
Sagiyama	2017	Multiple	22	Diff	3D	Manual	Outline	Structural	[73]
Saleh	2020	Multiple	104	RA	2D	Manual	Circular ROI	Diffusion	[74]
Schnapauff	2009	Multiple	30	Other	Unclear	Manual	Circular ROI	Diffusion	[75]
Singer*	2016	Multiple	17	Other	Multiple	Multiple	Multiple	Diffusion	[21]
Soldatos	2016	Multiple	23	RA	Unclear	Manual	Unclear	Diffusion	[76]
Sumi	2015	US	25	Diff	Unclear	Manual	Outline	Diffusion	[77]
Teo	2021	OS	15	RA	3D	Manual	Outline	Structural	[78]
Tian	2021	US	14	diff	Unclear	Manual	Unclear	Diffusion	[79]
Tong	2019	LMS	10	Other	Unclear	Manual	Outline	Diffusion	[80]
Uhl	2006	OS	8	RA	2D	Manual	Outline	Diffusion	[81]
Uhl	2006	OS	8	RA	2D	Manual	Outline	Diffusion	[82]
Valdes-Devesa	2019	Multiple	10	Diff	Unclear	Manual	Outline	Diffusion	[83]
Vossen	2008	LMS	10	RA	2D	Manual	Unclear	Diffusion	[84]
Wang	2013	OS	35	RA	Unclear	Manual	Outline	Diffusion	[85]
Wang	2017	OS	12	RA	2D	Manual	Circular ROI	Diffusion	[86]
Welzel	2018	CS	35	Diff	Unclear	Manual	Outline	Diffusion	[87]
Wu	2018	Multiple	22	Diff	MS	Manual	Outline	Diffusion	[88]
Xie	2019	US	29	Diff	3D	Manual	Outline	Diffusion	[89]
Xing	2018	Multiple	7	Diff	Unclear	Manual	Outline	Structural	[90]
Yakushiji	2009	Multiple	40	Other	Unclear	Manual	Outline	Diffusion	[91]
Yang	2016	Multiple	3	RA	2D	Manual	Outline	Diffusion	[92]
Yu	2021	Multiple	6	other	Unclear	Manual	Unclear	Unclear	[93]
Zeitoun	2018	OS	31	Diff	2D	Manual	Circular ROI	Diffusion	[94]
Zhang	2022	Multiple	41	other	2D	Manual	Outline	Diffusion	[95]

An overview of the different aspects of each segmentation strategy and its usage is shown in Table 2. For the segmentation method, drawing an outline was most common (49), compared to placing a circular ROI (22). Most studies performed single-slice segmentations (36), followed by segmentations on all slices (17) and multislice segmentation (7). Virtually all segmentations were performed manually (79) with only two strategies being semi-automated. Most segmentations were done on DWI data (70), with six strategies segmenting on structural images (T1/T2) and transferring to DWI data afterward. Most strategies segmented the whole tumor (39), or a specific area (22). Some strategies excluded necrotic, cystic, or hemorrhagic areas (15), and a few studies excluded the peripheral areas of the tumor (6). Some studies placed multiple, usually circular, ROIs (7).

**Table 2:** There is a high degree of variation in certain aspects of the segmentation strategy, such as the method, volume, and area. Other aspects, like the process and image modality, are more homogeneous

Property	Option	N
Method	Outline	49
	Circular ROI	22
	Unclear	10
Volume	2D	36
	Multislice	7
	3D	17
	Unclear	21
Process	Manual	79
	Semi-Automated	2
Image Modality	Structural	9
	Diffusion	70
	Unclear	2
Area	Excludes edge	6
	Excludes other	15
	Specific area	22
	Whole tumor	39
	Multiple ROIs	7
	Unclear	6

Table 2 additionally shows that a number of studies do not report all aspects of the employed segmentation strategy. We found the following frequencies of unreported details of the segmentation strategies: zero (55 studies), one (19 studies), two (4 studies), three or more (3 studies). The majority of included studies report all aspects we intended to analyze, 54/81. Nineteen studies did not mention one aspect and eight studies failed to report two or more aspects.

Out of the 76 articles, eight included one or more references to other works regarding segmentation strategies. These references provided background on the inclusion or exclusion of a region for segmentation (seven articles) and the definition of a region to be segmented (four articles).

Based on these results, six segmentation approaches were selected to be representative of the majority of studies listed in the review. These segmentation strategies, which will be compared in the next section, are defined as follows:

I. The outline of the whole tumor was drawn on the axial slice containing the largest tumor section (hereafter ‘Whole Tumor’).
II. The outline of the whole tumor was defined as in [I], but drawn 2 mm within the outer edge to avoid partial volume effects (hereafter ‘Inside’).
III. The outline of the whole tumor was defined as in [I], excluding any necrotic, cystic, or hemorrhagic areas (hereafter ‘Solid’).
IV. The outline of the whole tumor was drawn slightly within the outer edge to avoid partial volume effects excluding any necrotic, cystic, or hemorrhagic areas (hereafter ‘Inside Solid’).
V. A single circular ROI was drawn on the axial slice containing the largest tumor section, with the largest possible radius that does not exceed the tumor edges (hereafter ‘Circle’).
VI. Three circular ROIs placed in the most diffusion-restricted areas of the tumor were drawn on the axial slice containing the largest tumor section. The ROIs were drawn such as to maximize the total area within the diffusion-restricted component of the tumor but not exceed into the less restricted areas (hereafter ‘Max DWI circles’).

**Fig. 2:**
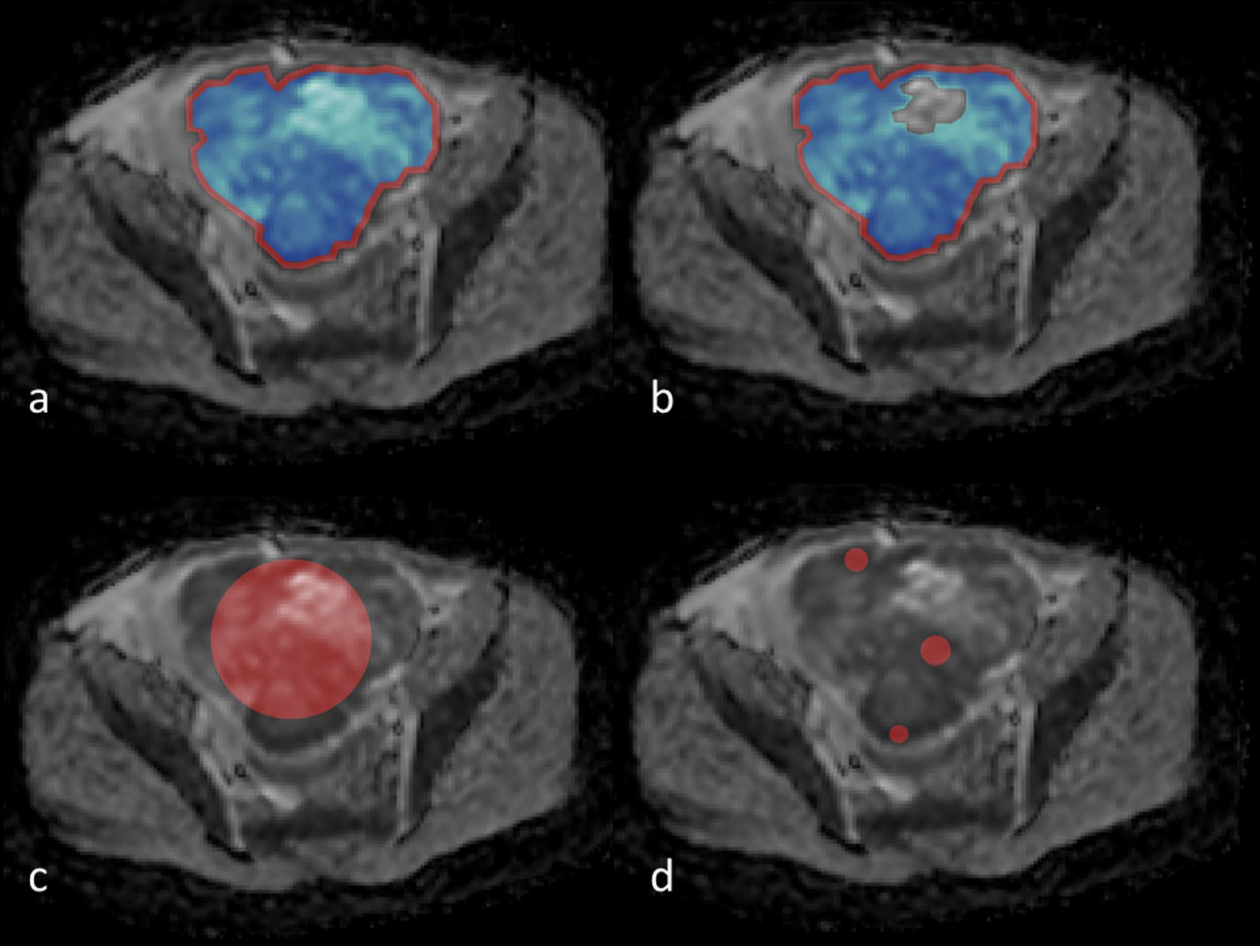
Illustrative example of diffusion-weighted image with regions of interest (ROIs) of six commonly used segmentation areas highlighted. **a** ‘Inside’ strategy, blue area. ‘Whole tumor’ strategy, red + blue area. **b** ‘Inside solid’ strategy, blue area. ‘Solid’ strategy, red + blue area. **c** ‘Circle’ strategy. **d** ‘Max DWI circles’ strategy

An example of these strategies is given in Fig. 2.

### Segmentation analysis

**Fig. 3:**
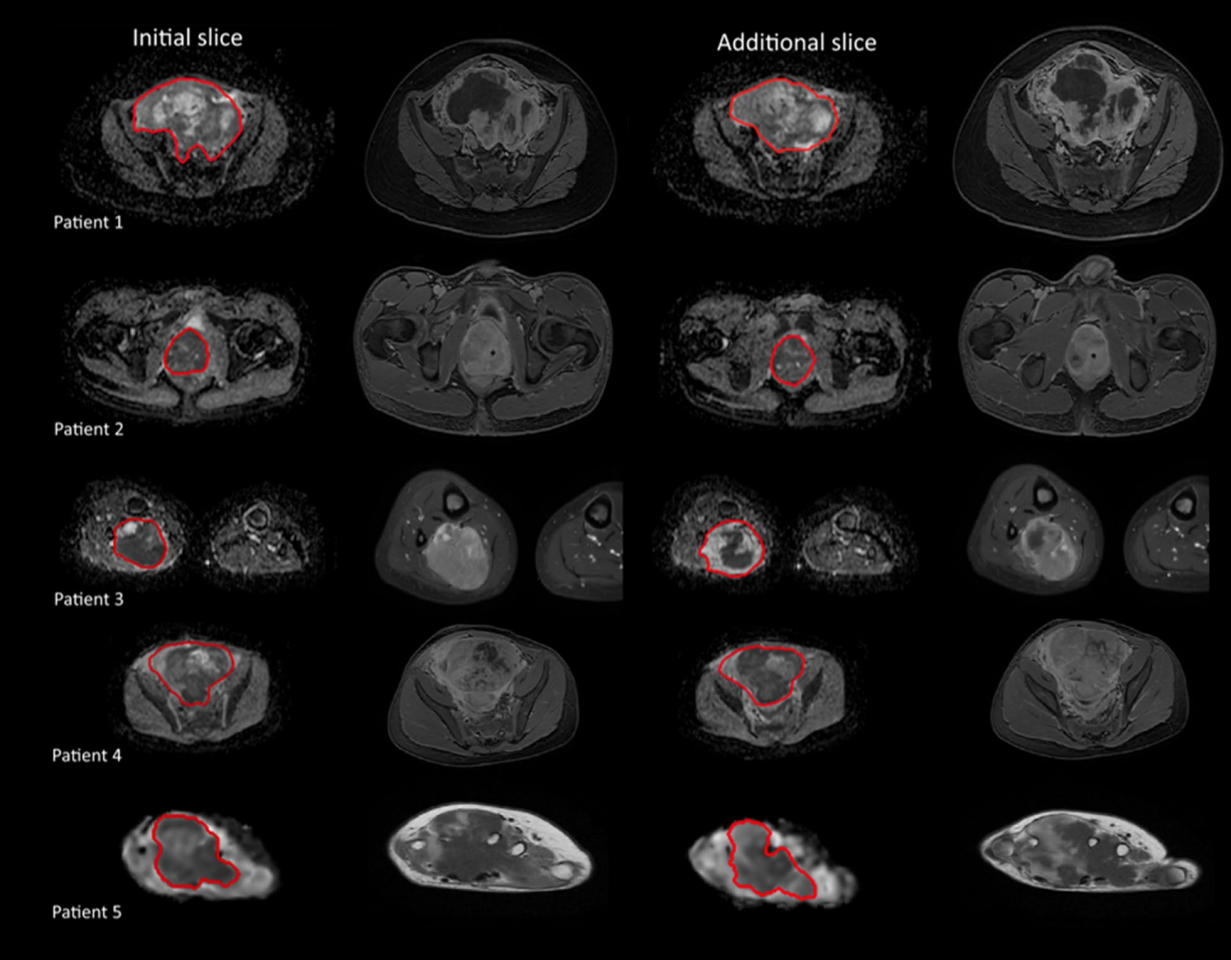
Apparent diffusion coefficient (ADC) maps and T1 post-contrast images for both the initial slice (columns 1 and 2) and the additional slice (columns 3 and 4) where segmentations have been made. The ADC maps include the outline of segmentation strategy 1 as described in Results

Figure 3 shows two example slices of the ADC and T1 post-contrast images for the considered patients. The percentage change between the mean ADC of all included voxels was calculated pairwise for all segmentation strategies. This is shown in Table 3. Excluding the edge yields a minor difference. Excluding necrotic, cystic, or hemorrhagic components results in a decrease in the mean ADC of around 6%. Placing a circular ROI instead of an outline yields a minor difference. Only measuring the most diffusion-restricted areas results in a decrease in the mean ADC of around 20%. The table furthermore shows whether the ADC distributions differ significantly as determined by the two-sample Kolmogorov–Smirnov test. Apart from the combinations of ‘Whole Tumor’—‘Inside’ and ‘Solid’—‘Inside Solid,’ all pairwise comparisons result in statistical differences.

**Table 3:** Percent difference of the mean apparent diffusion coefficient (ADC) averaged over all patients between every pair of strategies described in Methods

Method	Whole tumor (%)	Inside (%)	Solid (%)	Inside solid (%)	Circle (%)	Max DWI circles (%)
Whole tumor		0.50	**− 5.58**	**− 5.82**	**0.73**	**− 21.66**
Inside	− 0.50		**− 6.05**	**− 6.30**	**0.23**	**− 22.05**
Solid	**5.91**	**6.44**		− 0.26	**6.68**	**− 17.03**
Inside solid	**6.18**	**6.72**	0.26		**6.96**	**− 16.81**
Circle	**− 0.73**	**− 0.23**	**− 6.27**	**− 6.51**		**− 22.23**
Max DWI circles	**27.65**	**28.29**	**20.53**	**20.21**	**28.58**	

Bold numbers indicate significantly different distributions as determined by the two-sample Kolmogorov–Smirnov test. Excluding the edge from an ROI yields no significant differences in the distribution. All other methods do yield different distributions

**Fig. 4:**
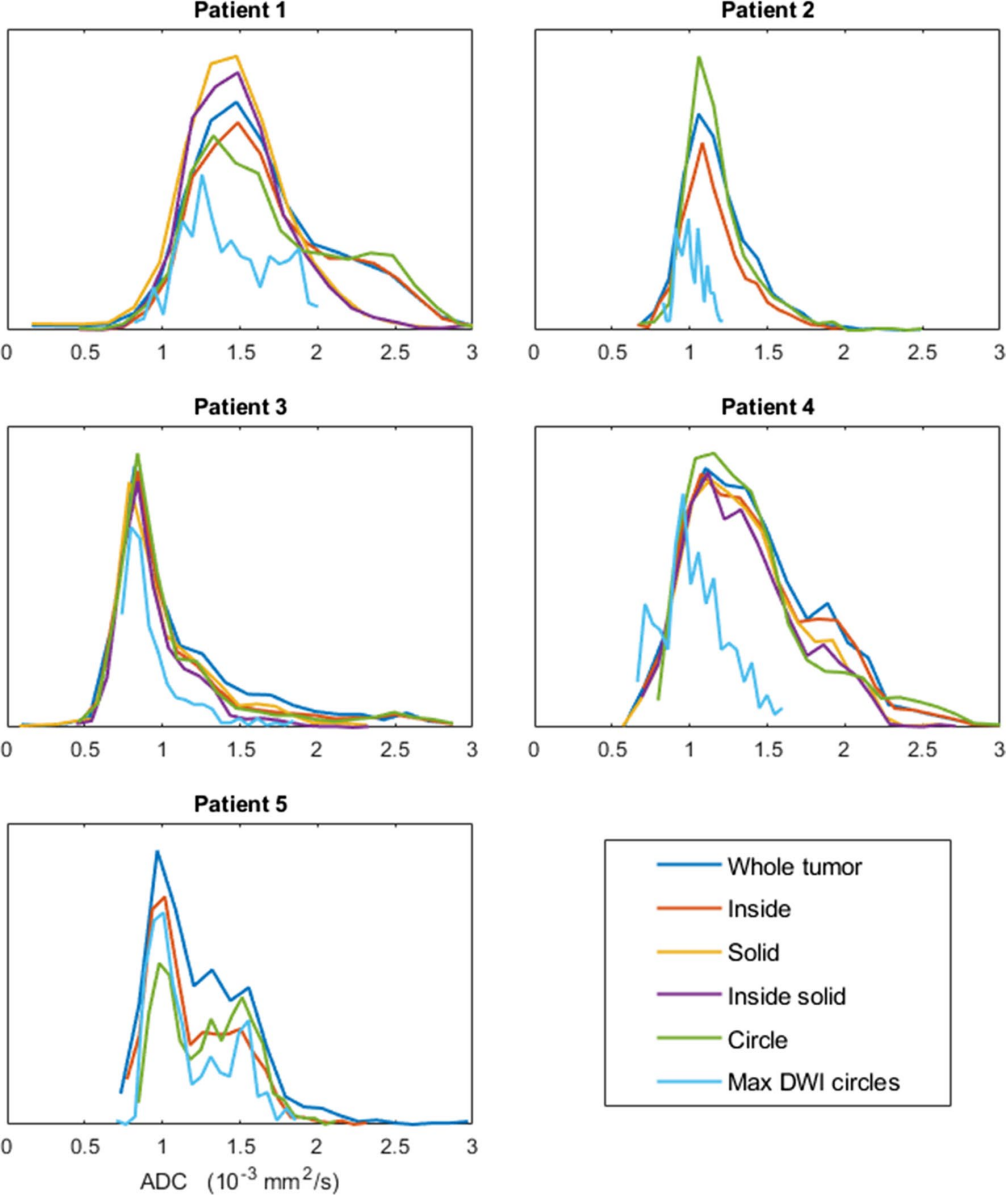
Normalized histograms of the apparent diffusion coefficient (ADC) of different regions of interest (ROIs) acquired with the strategies described in Methods. Varying segmentation methods can yield different distributions of the measured diffusion. Including necrotic, cystic, or hemorrhagic regions can result in non-Gaussian distributions. Patients 2 and 5 did not show any necrotic, cystic, or hemorrhagic tumor components and as such, specific ROIs that exclude these have not been drawn

Figure 4 shows the ADC distribution per patient for each of the six segmentation strategies. In patients where the tumor has a large necrotic component (1 and 4), there is a visible difference between strategies that exclude this component and those that do not. Not excluding the necrotic areas results in a small additional peak in the histogram.

**Fig. 5:**
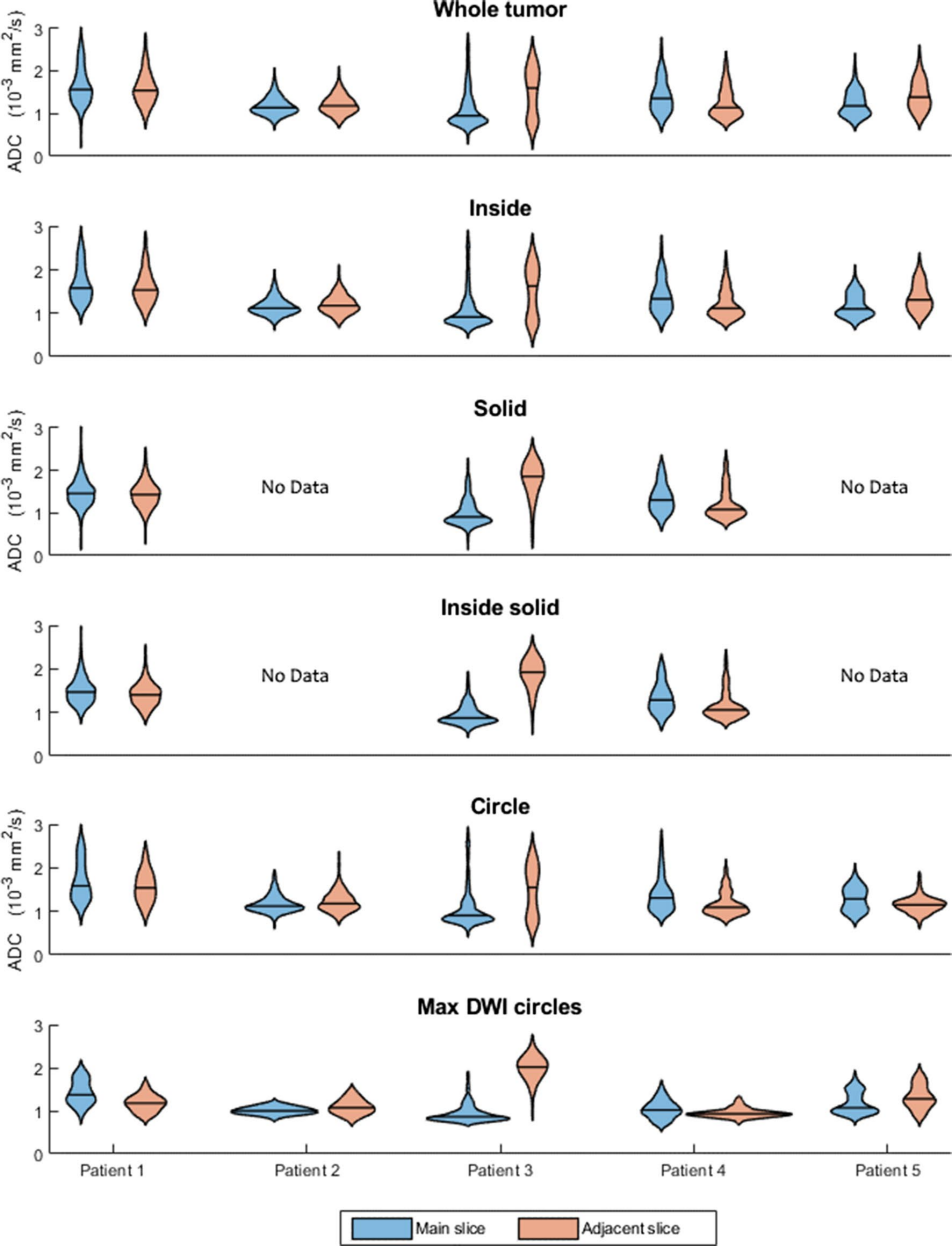
Violin plots of the apparent diffusion coefficient (ADC) estimated on two slices spaced two slices apart from each other. The estimations were derived from five patients with the regions of interest (ROIs) as defined by the six different segmentation strategies. Patients 2 and 5 did not show any necrotic, cystic, or hemorrhagic tumor components and as such, specific ROI that excludes these have not been drawn

Figure 5 shows the ADC distributions obtained by applying each segmentation strategy on two nearby slices for each patient. The distributions change visibly for patients 3–5 for each strategy. For patients 1 and 2, this difference is less clear from the shape of the distribution. When tested with the Kolmogorov–Smirnov test, there is a significant difference between the ADC distributions measured this way for all patients and all segmentation strategies.

The percentage change of the mean ADC of each tumor between two nearby slices averaged for all methods was small for patients 1–2 (4.55% and 5.42%), moderate for patients 4–5 (15.33% and 11.23%), and very large for patient 3 (96.0%). The values for all methods and patients are shown in Table 4.

**Table 4:** Percentage difference of mean ADC when measuring with the same strategy on a slice two steps down in the caudal direction. The measured mean ADC can drastically vary between nearby slices if the tumor is heterogeneous. No measurements were done with the solid strategy on patients 2 and 5, as these did not have any necrotic, cystic, or hemorrhagic components. All methods yield significantly different distributions as determined by the two-sample Kolmogorov–Smirnov test

Method	Patient (%)				
	1	2	3	4	5
Whole tumor	3.43	3.03	33.66	11.99	15.37
Inside	5.26	4.86	38.80	13.81	15.43
Solid	2.86		77.16	10.99	
Inside solid	5.59		98.58	13.31	
Circle	7.62	5.32	37.37	16.11	10.48
Max DWI circles	17.45	8.20	111.96	8.36	12.40
Mean	7.03	5.35	66.26	12.43	13.42

## Discussion

In this study, we investigate the effect of segmentation strategies on the computation of ADC values in patients with sarcoma. With a literature review, we show the existence of a large variety of methods that are applied in the field. Furthermore, we demonstrate with data from a small sample of pediatric rhabdomyosarcoma patients that applying different methods can significantly affect the measured ADC value.

### Literature review

Most prior investigations to date on the variability due to segmentation methods have focused on the effect of raters or algorithms [16, 17], even when extending the search beyond sarcomas. One notable study by Schurink and colleagues looked at both interrater differences as well as single- and multislice segmentations [18] and found that the segmentation volume affects most radiomics much more than the experience of the rater.

Our literature review shows that there is a consensus between most studies for performing the segmentation on DWI and not on structural images, as well as a clear preference for manual segmentation as opposed to semi-automated methods. Conversely, no consensus or even established strategy could be determined regarding the segmentation method (outline or circular), the area inclusion, and the segmentation volume.

Examining the reason for such a diversity of strategies is difficult given that segmentation approaches are often presented without any supporting evidence or rationale for their choice. We find that only eight out of the 76 included studies refer to previous literature to justify some aspects of their methods. It therefore remains unclear whether any particular strategy was chosen based on its effectiveness or its ease of implementation. Furthermore, many specific aspects needed for reproducibility are sometimes either not reported well or not at all. Out of all included studies, 27 did not report all aspects of the segmentation applied. A further 20 were excluded from the review altogether for not reporting any details. This large degree of variation highlights a need for a standard for reporting details on tumor segmentation, in analogy to other established radiological practices [19].

Altogether, when looking at segmentation strategies, we find a lack of reported details, a large variability of literature, and a lack of evidence-based strategies. This suggests that segmentation might often be an afterthought in study design.

### Segmentation analysis

When comparing different segmentation strategies for estimating ADC values from pediatric rhabdomyosarcoma cases, we find that the choice of segmentation area can affect the measured ADC. Most notably, excluding necrotic, cystic, and hemorrhagic areas results in a decrease of 5.6% in the mean ADC. It furthermore results in an ADC distribution that is more Gaussian than when including such regions. Next, measuring only the most diffusion-restricted parts of the tumor results in a decrease of 21.6%. The resulting segmentation contains too few voxels to create a useful ADC distribution. Additionally, drawing a circular ROI instead of an outline results in a small, but still significant, difference in ADC (< 1%). Finally, excluding the most peripheral areas of the tumor does not result in any significant differences to the mean ADC or ADC distribution.

Varying the slice on which segmentations are made has a much larger effect, especially for heterogeneous tumors. When looking at all patients and all strategies, the mean ADC averaged a 26.5% difference when measured on a nearby slice. These results are in line with results described by Guo and colleagues [20], who compared 2D and 3D ROIs on 56 patients with breast phyllodes tumors. They found that single-slice segmentations generally resulted in a higher ADC. Similarly, Singer and colleagues [21] compared four different segmentation strategies on data of 22 patients with soft tissue lesions. They showed that whole-tumor segmentations resulted in lower ADC values compared to single-slice and multislice segmentations. Such a large difference in mean ADC measured across nearby slices is striking when considering that more than half of the included studies are using single-slice segmentations. As this method can produce strongly varying results—especially in heterogeneous tumors—care should be taken when using it and preference should be given to methods annotating multiple slices, where possible.

For pediatric rhabdomyosarcoma specifically, it would be useful to further investigate the effect of different segmentation strategies. As the disease is rare, any sufficiently powerful study requires a multicentric design. Since such a design typically already entangles variability in terms of acquisition hardware and protocols, better understanding the additional variance introduced by different segmentation methods could help maximizing statistical power in this field.

### Considerations

Rhabdomyosarcoma is the primary focus of this review. As an insufficient number of studies in this field described the segmentation approach with sufficient detail, the search was expanded to include other types of soft tissue sarcoma, including ones only arising in adults. As these other types of sarcomas might exhibit different radiological manifestations, segmentation strategies used in their study might differ from those used in rhabdomyosarcoma.

Another limitation of our study is that only five patients were included in our segmentation analysis. This small number might bias our results toward individual properties and may not represent the ‘average’ patient with rhabdomyosarcoma. However, even with such limited sample size we are able to demonstrate that certain aspects of the segmentation strategy can affect ADC measurements, showing proof of concept of the importance to design and report tumor segmentation strategies. Finally, we only assessed the impact of the measurement site and method on the initial measurement and not on the impact on tumor response assessment.

## Conclusion

There is a large variation in segmentation strategies for sarcomas. This variation is difficult to characterize, as many articles do not justify why a strategy is chosen. Details of the segmentation method are furthermore often not well reported or not reported at all. When comparing strategies on our own data, we find that ADC estimates derived from a single slice depend highly on their location within the tumor. For this reason, using multislice or full-volume segmentations is preferred.

We call upon researchers to include clear imaging and reporting guidelines in protocols of prospective multicenter studies. It is our advice to keep in mind how the segmentation strategy might affect any computations made and to report all of the important details for making segmentations reproducible.

## Supplementary Information

**Additional file 1: **Search terms used in PubMed literature search on 24-03-2022.

## Data Availability

The datasets analyzed during this study are available from the corresponding author on reasonable request.

## Abbreviations

ADC: Apparent diffusion coefficient
DWI: Diffusion-weighted imaging
MRI: Magnetic resonance imaging
ROI: Region of interest
