# Supplementary material for: Methodological considerations on segmenting rhabdomyosarcoma with diffusion-weighted imaging—What can we do better?
Source: Insights Imaging. 2023 Jan 31;14:19. doi: 10.1186/s13244-022-01351-z (PMC9889596; doi:10.1186/s13244-022-01351-z)
Supplement: Supplementary file 1 — Additional file 1: Search terms used in PubMed literature search on 24-03-2022. [file 13244_2022_1351_MOESM1_ESM.docx]

# Supplementary

## Search terms

Diffusion Magnetic Resonance Imaging"[Mesh] OR "Diffusion Tensor Imaging"[Mesh] OR "Whole Body Imaging"[Mesh] OR apparent diffusion OR apparent diffusion coefficient OR diffusion weighted MRI OR diffusion weighted MR OR dynamic contrast enhanced OR dynamic contrast enhanced MR OR dynamic contrast enhanced MRI OR diffusion magnetic resonance imaging OR dynamic contrast enhanced MR perfusion OR MR-DCE OR perfusion-weighted imaging OR diffusion weighted sequences OR diffusion restriction OR diffusion tensor imaging OR advanced MR imaging OR advanced magnetic resonance imaging OR whole-body MR OR whole body MRI AND Sarcoma
